# Sickle cell disease: suspect, check, diagnose—practical tips for non-SCD experts to suspect and diagnose SCD in low-prevalence European settings

**DOI:** 10.3389/fmed.2025.1646682

**Published:** 2025-09-16

**Authors:** Giovanni Palazzi, Silvia Benemei, Cristiano Gallucci, Francesco Arcioni, Silverio Perrotta

**Affiliations:** ^1^U.O. Oncoematologia Pediatrica, Azienda Ospedaliero-Universitaria di Modena, Modena, Italy; ^2^Medical Affairs, Pfizer, Rome, Italy; ^3^Pediatric Oncology-Hematology, Azienda Ospedaliera di Perugia, Perugia, Italy; ^4^Dipartimento della Donna, del Bambino e di Chirurgia generale e specialistica, Università degli Studi della Campania "Luigi Vanvitelli", Napoli, Italy

**Keywords:** sickle cell disease, diagnosis, medical history, illness script, diagnostic tools, clinical script

## Abstract

Sickle cell disease (SCD) is the most common monogenic disorder, including a group of autosomal recessive hemoglobinopathies characterized by hemoglobin polymerization and sickling of red blood cells when low oxygen concentrations are present. SCD has a growing public health significance, affecting nearly 8 million people globally, with a high prevalence observed in Sub-Saharan Africa and Mediterranean countries. Improved understanding of SCD is essential, particularly given recent migratory flows that have contributed to an increase in the number of affected individuals in Europe and Italy. An early diagnosis is crucial to start the appropriate therapy to ensure the patients with the best outcome and improved quality of life, but clinical signs of SCD are often not easily recognized as symptoms are nonspecific and difficult to frame within the context of a congenital hemolytic disease. Given the availability of simple and multiple diagnostic tools, a simplified approach based on red-flags can facilitate the diagnostic suspicion in clinical practice to promptly identify individuals to be referred to specialized centers. The present narrative review aims to discuss the main clinical features, diagnostic tools of SCD, and provide practical illness scripts to facilitate the approach of non SCD-expert healthcare professionals to its diagnosis. Patient’s history, including ethnicity, region of origin, familial cases of SCD and other congenital or unexplained anemias, previous clinical manifestations, remain fundamental in guiding diagnostic suspicion of SCD, together with a few crucial lab parameters. The implementation of screening projects is essential to ensure early diagnosis and rapid access to care for affected individuals.

## 1 Introduction

Diagnosis is the cornerstone of providing safe, efficient, and effective medical care. A physician’s ability to diagnose a patient’s illness—that is, arrive at an explanation for a patient’s health problem—is one of the hallmarks of medical expertise and is fundamental to assigning correct and effective treatments. The most promising and effective way to improve the outcomes of the diagnostic process is to improve the education of health professionals (1).

Despite increased attention paid to sickle cell disease (SCD) and the presence of national guidelines and recommendations, the awareness of clinical suspicion signs and diagnostic tools may still be improved among non SCD-dedicated physicians, that are often first-line care providers, including but not limited to emergency room (ER) physicians, internists and pediatricians (2). Prenatal screening is not available in every country, contributing to diagnostic and referral delay (3). In Italy only a small number of local newborn screening programs for SCD have been reported, including those launched in Modena, Ferrara, Novara, and Pordenone, following the presence of carriers due to immigration (4), the pilot screenings in Padova and Monza (5), the protocols for the screening of carriers for thalassemia and hemoglobinopathies in women between 13 and 50 years in Sicily (6, 7), and early diagnosis project involving the pediatricians in Campania (8), which showed that screening using point-of-care tests by primary care is feasible and effective for early detection in at-risk children (9). Unfortunately, some of these programs were discontinued due to lack of funding or the end of the project and Italy still remains without a universal screening campaign (10). This discontinuity may contribute to delayed diagnoses, missed early intervention, and an increased risk of preventable complications among affected individuals. Avoidable delays are the most burdensome consequences of the not optimal knowledge of the disease and its diagnostic management. Importantly, the availability of multiple diagnostic tools for SCD (4, 11), and their relative appropriateness to different clinical settings may be a source of complexity.

This scenario suggests that a simplified approach may be helpful in clinical practice for non-SCD experts. It has been suggested that physicians can enhance their diagnostic performance establishing new connections between their knowledge and specific clinical encounters, thereby enabling stronger associations between clinical features and the knowledge retained in memory (1, 12). For instance, clinicians often rely on a mental model developed through experience to efficiently recognize and reason through medical conditions. This model can be defined as the illness script, a structured model that helps to retrieve clinical knowledge during diagnosis. According to Bowen’s model, a main step in the diagnostic reasoning is the definition of “illness scripts” developed by physicians, based on their exposure to patients, and include key clinical elements that help prompt diagnostic suspicion (12).

The present narrative review aims to discuss the SCD clinical features with a critical approach leading to an SCD script, to facilitate the retaining of those features in mind, thus increasing the confidence in suspecting a SCD diagnosis when appropriate. To this aim we will present a practical SCD script applicable to adults and children, for both acute and chronic setting, considering it a possible daily practice tool for non-SCD experts.

We will provide a clinical-oriented overview, aligned with current guidelines and including diagnostic approaches and tools, to help non-SCD expert physicians for a timely suspicion of SCD and diagnostic reasoning, hence increasing the rate of diagnosis and referral.

## 2 Sickle cell disease: pathophysiology and global prevalence

**Figure 1:**
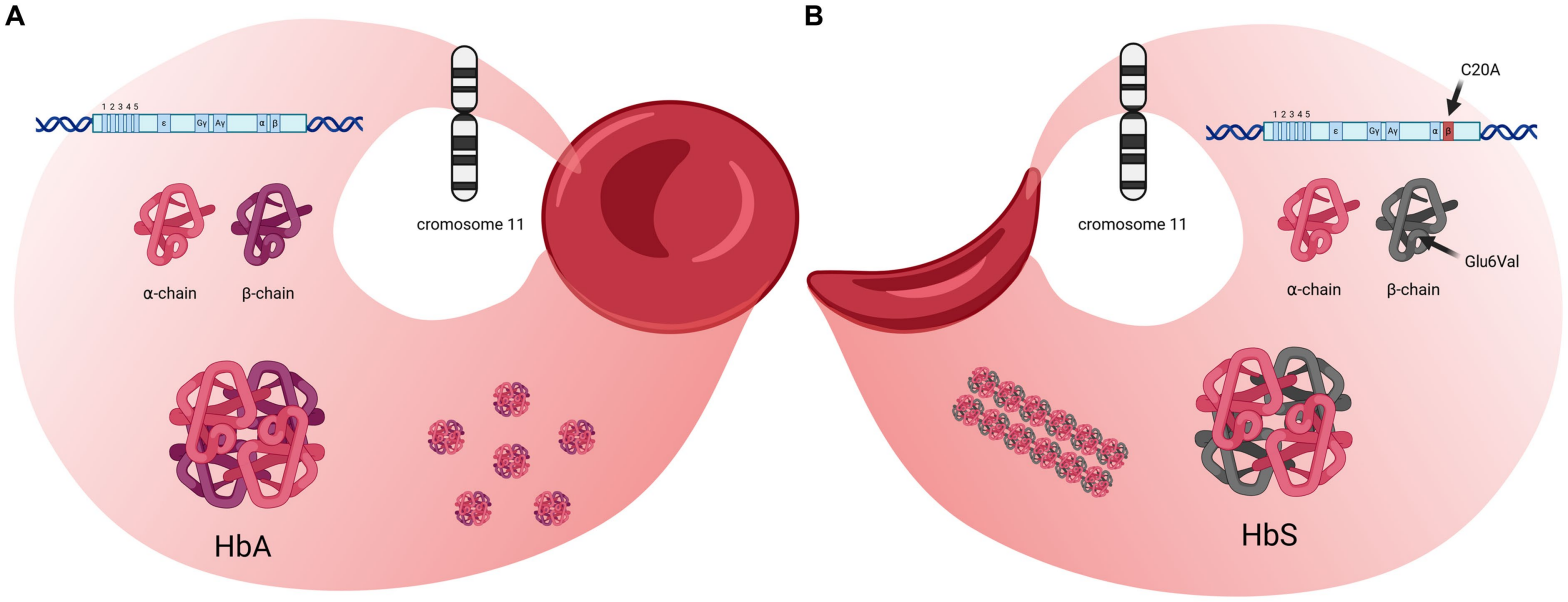
Molecular and structural differences between normal hemoglobin (HbA) and sickle hemoglobin (HbS). **(A)** structure and subunits of HbA found in healthy red blood cells. The HbA molecule consists of two *α*-chains and two *β*-chains, assembled into a globular structure, allowing red blood cells to maintain a round, flexible shape for efficient oxygen transport. The genetic sequence on chromosome 11 that encodes the *β*-chain is shown, with no mutations present. **(B)** Structure and impact of sickle hemoglobin (HbS) which differs from HbA because of a substitution of valine for glutamic acid at position 6 (Glu6Val) in the *β*-chain. This single-point mutation leads to hemoglobin polymerization under low-oxygen conditions, causing red blood cells to adopt a rigid, sickle shape. These deformed cells can obstruct blood flow. The genetic sequence on chromosome 11 highlights this mutation (C20A). Created in BioRender (2024) https://BioRender.com/n07g051.

Sickle cell disorders are a group of monogenic conditions that include the most common hemoglobinopathies worldwide, together with thalassemia, inherited in an autosomal recessive fashion (13). Homozygosity for the abnormal hemoglobin variant S (HbS) is the most common type of SCD and causes the most severe clinical phenotype, termed sickle cell anemia (Figure 1); however, patients with SCD may display other genotypes due to combination of the HbS gene with other non-HbS variants such as HbC, HbE, and HbD or to the many types of HbS-*β*-thalassemia (14). The severity of SCD can vary depending on the patient’s genetic background, as different Hb variants influence the polymerization of HbS. The residual expression of fetal Hb (HbF) in postnatal red blood cells (RBCs) can interfere with the polymerization of HbS, reducing the tendency of RBCs to sickle. Moreover, the simultaneous presence of mutations in the alpha-globin gene (alpha-chain defects) in patients with HbS, can attenuate the clinical manifestations and severity of SCD again through polymerization interference (13, 15). The inheritance of both normal adult HbA and HbS is defined as sickle cell trait (HbAS) (16), and provides a survival advantage against death from malaria, thus explaining the higher incidence of SCD in countries where this mosquito-borne disease is particularly widespread (17). The sickle cell trait usually does not trigger a clinical phenotype except under extreme hypoxic conditions (18).

SCD has a large and growing global public health significance. According to the latest estimates, the number of newborns with SCD increased from 453,000 to 515,000 between 2000 and 2021, likely attributed also to improvements in diagnosis (3). Of these births, 76.5% were of the HbSS and HbS*β*^0^ genotypes. All-age global SCD prevalence raised by 41.4% in 2021, with 7.74 million cases (3). The highest SCD disability burden is currently concentrated in western and central sub-Saharan Africa, Mediterranean area and India (3). In Europe, the prevalence of the disease is heterogeneous, from 0.4 (range 0.3–0.5) to 18.8 (16.5–21.1) per 100,000 population in Netherlands and Greece, respectively (3). In Italy, the prevalence of 4.4 (3.8–5.0) per 100,000 general population has been estimated in 2021, but is reported to be much higher in the subset of sub-Saharan origin (3, 19, 20). Recent real-world data indicate that the Italian SCD population comprises two distinctly different subgroups: the native SCD patients, whose more frequent genotype is HbS*β*, and non-native SCD patients, mainly Africans or Afro-Americans whose more frequent genotype is HbSS (20).

Patients with SCD have on average a reduced life expectancy compared to the general population. Although this may vary depending on individual factors and healthcare access, estimates suggest a reduction of up to 20 years in some high-income settings, with an even greater gap in low-income countries. This difference is likely due to more difficult in access to medical services in low-income countries. In middle- and high-income countries, improved survival has been achieved through early diagnosis via newborn screening, prophylactic antibiotics, hydroxyurea therapy, and accessible care programs (13, 21).

Early diagnosis of SCD allows for risk mitigation and early treatment intervention. Universal newborn screening programs for SCD are currently available only in some countries worldwide, including USA. For what concerns Europe, the screening map is quite heterogeneous, with some countries, including Italy, not yet offering a universal or targeted newborn screening program for SCD, despite recommendations of national guidelines (22, 23).

## 3 Main complications, characteristics, and timing in SCD

The morbidity of SCD is progressive throughout the life span. SCD complications can have an acute onset or a chronic progression, with exacerbations of chronic complications. In addition, some SCD complications are more frequent at specific life stages, while others can occur across multiple life stages or persist throughout life (21). Besides the reduced life expectancy, the quality of life is also poor in this patient population (14, 24). Tables 1 and 2 summarize some of the most frequent acute and chronic complications that could arise in pediatric and adult patients presenting to the ER or to healthcare assistance and that could be suggestive of undiagnosed SCD.

**Table 1:** Main potential acute clinical manifestations of SCD in pediatric and adult patients and appropriate diagnostic tools and parameters.

Manifestation	Cause	Features	Diagnostic approach	Ref
ACS	Pulmonary infiltrate caused by *Streptococcus pneumoniae*, *Mycoplasma pneumoniae* and *Chlamydia pneumoniae* Unknown cause in some cases	Adults: chest pain, shortness of breath, neurologic findings, and pain preceding ACS Children: wheezing and fever, with pain being less common Fever, leukocytosis, pleuritic chest pain, pleural effusion, cough Development during the first 3 to 4 days of an acute pain episode Rarely progressing to multiorgan failure syndrome in addition to respiratory failure	Complete lab investigations, blood gas analysis, ECG and thorough physical inspection, imaging tests Defined as the presence of new infiltrate at imaging together with at least one of the following symptoms: fever, chest pain, dyspnea, desaturation	(21, 22, 25, 37–40)
Acute splenic sequestration/acute anemia	Splenic sequestration of sickled RBCs	Common in children <5 years of age who have a spleen not yet autoinfarcted Left upper quadrant pain with a rapid splenomegaly, leukopenia, thrombocytopenia, increased reticulocyte counts and rapidly decreasing Hb Can be associated with an ACS episode or viral/bacterial infection	Medical history, evaluation of clinical presentation and complete lab investigations, blood culture	(21, 22, 38)
Acute recurrent pain-VOC	Microvascular obstruction leading to impaired oxygen supply to the periphery and ischemia–reperfusion injury, oxidative stress, inflammation, endothelial dysfunction	Sudden pain onset, sharp and throbbing, lasting up to 6–7 days before resolving Common in the lower back and extremities Pain crises can begin as early as the first year of life Variable number of VOCs and pain crises during lifetime (up to 18.2 VOCs/year) Common in women during pregnancy Triggered by patient features (DNA variants of Hb, ethnic group) and environmental factors (hypoxia, dehydration, cold, prolonged immobility, infection, fever, acidosis) Possible clinical consequences are ACS, hepatic and renal involvement, and cerebrovascular accident	Medical history (including site, intensity and kind of pain and other associated symptoms), lab and imaging investigations, also aimed to rule out any possible surgical emergency Attention to all patient-reported information, including perceived changes in functional capacity and overall quality of life	(22, 38, 41–43)
Infections	Functional asplenia contributes to the severe infections from *S. pneumoniae*, *Haemophilus influenzae*, *Neisseria meningitidis*	Fever in children can indicate infection Invasive infections, including bacteremia, sepsis, meningitis, pneumonia, and osteomyelitis, are the major cause of morbidity and mortality in children	Medical history (including vaccination and antibiotic prophylaxis) and ethnical origin, rapid evaluation of clinical presentation (fever) and complete lab investigations, blood count and culture	(16, 21, 22)
Osteomyelitis	Inflammation of bone secondary to *Staphylococcus aureus* infection	Childhood onset In the acute phase, almost indistinguishable from VOC, as in both cases symptoms are fever and a painful, swollen limb with limited motion Usually, osteomyelitis affects the diaphysis of long bones, but it is possible the involvement of any other bone	Complete lab investigations; culture from a sample of either bone, synovial fluid, or blood, to be integrated by radiological data	(44)
Priapism	Circulation obstruction in the microvessels of the corpora cavernosa	Painful erection of more than 4 h Can be either stuttering or recurrent It occurs more frequently between 20 and 50 years of age	Doppler ultrasound, blood gas analysis, intracavernous pressure have been used to better define the prognostic factors	(21, 22, 45, 46)
Stroke	Sickle cell-related vasculopathy of major cerebral arteries or complete occlusion	Ischemic stroke occurs in approximately 10% of children with SCD Hemorrhagic stroke is more common in young adults with SCD Stroke symptoms in children can be vague and should be carefully evaluated	Evaluation of acute neurologic symptoms Neuro-imaging (MRI, non-contrast CT, angio-MRI) should be quickly performed in case of acute neurological symptoms Non-contrast CT can rule out hemorrhagic stroke and may show ischemic stroke; CT scan might not detect ischemic stroke within the first 6 h	(21, 22, 38)
Venous thromboembolism	Endothelial dysfunction, hemolysis, increased levels of procoagulant factors, VOC	Developed in both children and adults Central venous lines, chronic kidney disease, history of stroke, and admission to intensive care (associated to increased immobility, systemic inflammation and a generally procoagulant state) can be associated with this disorder	Medical history, physical examination (pain, swelling, warmth, or discoloration), laboratory tests, and imaging studies	(21)

ACS, acute chest syndrome; CT, computed tomography; ECG, electrocardiogram; Hb, hemoglobin; Htc, hematocrit; MRI, magnetic resonance imaging; RBC, red blood cells; SCD, sickle cell disease; VOC, vaso-occlusive crisis.

**Table 2:** Main potential chronic clinical manifestations of SCD in pediatric and adult patients and appropriate diagnostic tools and parameters.

Description	Cause	Features	Diagnostic approach	References
Avascular necrosis	Vaso-occlusion leading to infarction of the articular surfaces and head of long bones	More common in young adults (50% of the patients by 35 years) Involves the hips, shoulders, the knee, the small joints of the hands and spine May be associated with eventual bone collapse Persistent local bone pain and limited motion of affected joints	MRI	(16, 47, 48)
Chronic anemia	Persistent decrease of Hb levels due to repeated hemolysis, with compensation sign	Decreased Hb levels Increased hemolysis indexes Increased reticulocytes Sickle cells	Complete blood investigation including reticulocyte count, LDH and bilirubin, peripheral blood smear	(11, 49)
Chronic pain	Bone infarction, chronic osteomyelitis, leg ulcers, avascular necrosis of the femoral or humeral head	Present on most days over 3–6 months in a single or multiple locations Common in adult patients (40% prevalence) Nociceptive, neuropathic, and central components affecting quality of life Sensation of pins and needles, hyperalgesia, and allodynia and described as numb, tingling, lancinating	Multidimensional assessment (location, type, and pattern of pain), functional limitations patient reported outcomes	(21, 43, 50)
Gallstone disease	Increased hemolysis	Onset in childhood and adolescence, frequent in adults Secondary to chronic hyperbilirubinemia	Abdominal ultrasound	(21, 51)
Leg ulcers	Vaso-occlusion and hypoxia of the skin High hemolytic rate, low Hb May develop without injury or underlying infection	More frequent in adults More frequent in the skin around the ankle, less common in the dorsum of the foot and the anterior tibia	Clinical evaluation, physical examination, laboratory testing	(16)
Ophthalmic disease	Sickling of erythrocytes and increased blood viscosity in HbSC individuals (higher Hb levels) leads to retinal ischemia and scarring Peripheral retinal arteriolar occlusions	Proliferative retinopathy, retinal detachment, vitreous hemorrhage, mainly in young adults but described also in children and adolescents Peripheral retinopathy is more frequent in HbSC and HbS/*β*^+^ than in HbSS and HbS/*β*^0^ patients	Ophthalmic evaluation	(16, 51)
Pulmonary hypertension	Increased pulmonary pressure (≥25 mmHg), likely due to increased blood viscosity, intravascular hemolysis inducing vascular dysfunction	Mainly in adults Left and right ventricular dilation and diastolic dysfunction Dyspnea, fatigue, increased morbidity and mortality	Right heart catheterization Non-invasive estimation by Doppler echocardiography	(16, 52, 53)
Renal dysfunction	HbS polymerization in the renal medulla	Childhood onset Hyposthenuria, nocturia and enuresis In adolescence frequent renal ischemia, hematuria proteinuria, and hyperfiltration Increased GFR, combined with glomerular hypertrophy results in glomerulosclerosis, and progresses chronic kidney disease and end-stage renal disease in the adult age Increased blood pressure	Evaluation of GFR and proteinuria	(16, 52, 54)

Hb, hemoglobin; HbS, hemoglobin S; HbSC, heterozygous for hemoglobin S and C; HbSS, homozygous for HbS; MRI, magnetic resonance imaging; GFR, glomerular filtration rate.

## 4 Facilitating SCD diagnostic suspicion

Non-SCD dedicated physicians, including ER physicians, may play a critical role in early detection of undiagnosed SCD and prompt treatment of its complications. It is a pleonasm saying that this critical role has its roots in the diagnostic suspect and is realized by a diagnostic plan (25).

**Figure 2:**
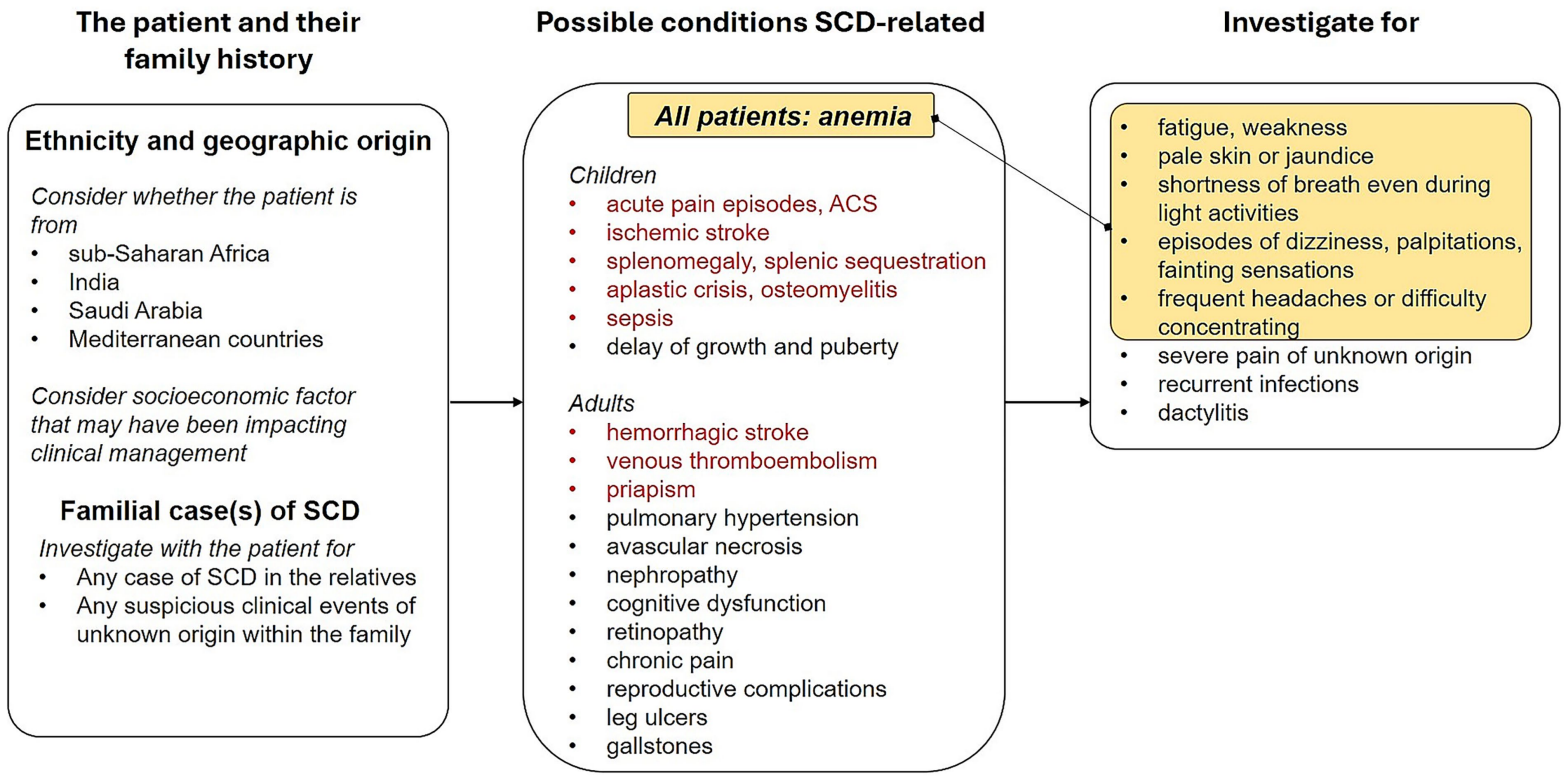
Script for diagnostic suspect of sickle cell disease in both pediatric and adult patients.

Here, we propose SCD illness scripts, summarizing the predisposing conditions, the pathophysiological insult and the clinical consequences of SCD, in children and adults both in the acute and chronic setting. The SCD scripts aim to facilitate physicians recalling SCD as a possible diagnosis when encountering some clinical features. If SCD scripts are kept in mind, all the following information would be easily recalled and considered in the diagnostic process (Figure 2).

### 4.1 Ethnicity and origins

Particular attention should be paid to patient’s ethnicity and geographic origin, considering the SCD is particularly common among people whose ancestors come from western and central sub-Saharan Africa, India, Saudi Arabia and Mediterranean countries and that migration and mobility increased the frequency of this genetic condition also in the American and European continents (3). In the Italian population, two phenotypes of patients with similar proportions have been identified by a retrospective study involving 34 hemoglobinopathies centers: children of African descent, typically HbSS, and adults of Caucasian descent, that are most frequently HbS/*β* genotype (20).

### 4.2 Family history

The presence of any familial case of either diagnosed SCD or clinical events ascribable to SCD and thalassemic trait should be investigated as well in case of a diagnostic suspect of this hemoglobinopathy, considering that SCD is an autosomal disorder with a recessive Mendelian inheritance requiring both parents to carry the Hb mutation (26).

### 4.3 Information about clinical conditions possibly due to SCD

The collection of information about frequency and timing of complications during a patient’s life course in a comprehensive history intake is no doubt helpful in SCD diagnostic pathway. Importantly, a huge number of varied conditions may emerge from the archetypal SCD pathogenic triad: red blood cell sickling, hemolysis and vaso-occlusion. Regarding the timing of SCD complications during the life course, acute pain episodes (due to vaso-occlusive crises, VOCs), acute chest syndrome (ACS) and ischemic stroke can occur at any life stage, although more frequent in younger patients. Importantly, SCD patients can exhibit a delay of growth and puberty during childhood and adolescence due to ongoing hemolytic anemia and due to the increased metabolic demands. In adulthood, hemorrhagic stroke, leg ulcers, pulmonary hypertension and reproductive complications can occur more frequently. Aplastic crisis, osteomyelitis, splenic sequestration and infarction and sepsis are more frequent in childhood and adolescence, while avascular necrosis, cognitive dysfunction, chronic pain, priapism, nephropathy, retinopathy, gallstones, and venous thromboembolism are generally more frequent in adulthood (Table 1) (21). Vaso-occlusion, with associated ischemia, is also responsible for ACS and avascular necrosis, while hemolysis-related endothelial dysfunction underlies also pulmonary hypertension, priapism, stroke and leg ulcers (21).

### 4.4 Clinical examination

With particular reference to pediatric patients, specific elements to be considered during physical exam are pallor (assessed by examining the palms of the hands and mucous membranes in non-Caucasian individuals), jaundice, and splenomegaly (less frequent with increasing age) (22). Attention should be paid to recurrent episodes of unexplained, atraumatic severe pain (in particular bone, abdominal and wandering pain), chronic hemolytic anemia, recurrent infections (mainly lung and bone infections), priapism episodes, and dactylitis characterized by painful swelling in the fingers and toes, all symptoms possibly consequent to sickling and hemolysis of RBCs (21, 22). Some clinical manifestations, including aplastic crisis, osteomyelitis, stroke, splenic sequestration and bronchopneumonia, could be indicative of SCD onset in children. The detection of hemolytic anemia (elevated LDH and bilirubin, reduced haptoglobin) in association with nonspecific complications (systemic inflammatory disease, pancreatitis, septic arthritis) should raise a stronger suspicion of SCD, considered that these manifestations are not pathognomonic signs of SCD, and differential diagnoses should be considered as well (22).

### 4.5 Variability

One of the major challenges in SCD diagnosis is the clinical variability of this disease. Despite the hereditary pattern, the severity and age of onset of SCD clinical manifestations vary among patients, under the influence of a combination of genetic, epigenetic and environmental factors (16, 27), including the genotype of globin genes and the presence of genetic polymorphisms that act as modifiers on globin genes (13, 16, 21, 27). Another relevant determinant, is the socioeconomic factor that, intertwined with the geographic area of origin and, hence, with the genetic background, influences the access to diagnosis and treatment (3) therefore modulating the clinical features.

## 5 Diagnostic approaches and tools for SCD

**Figure 3:**
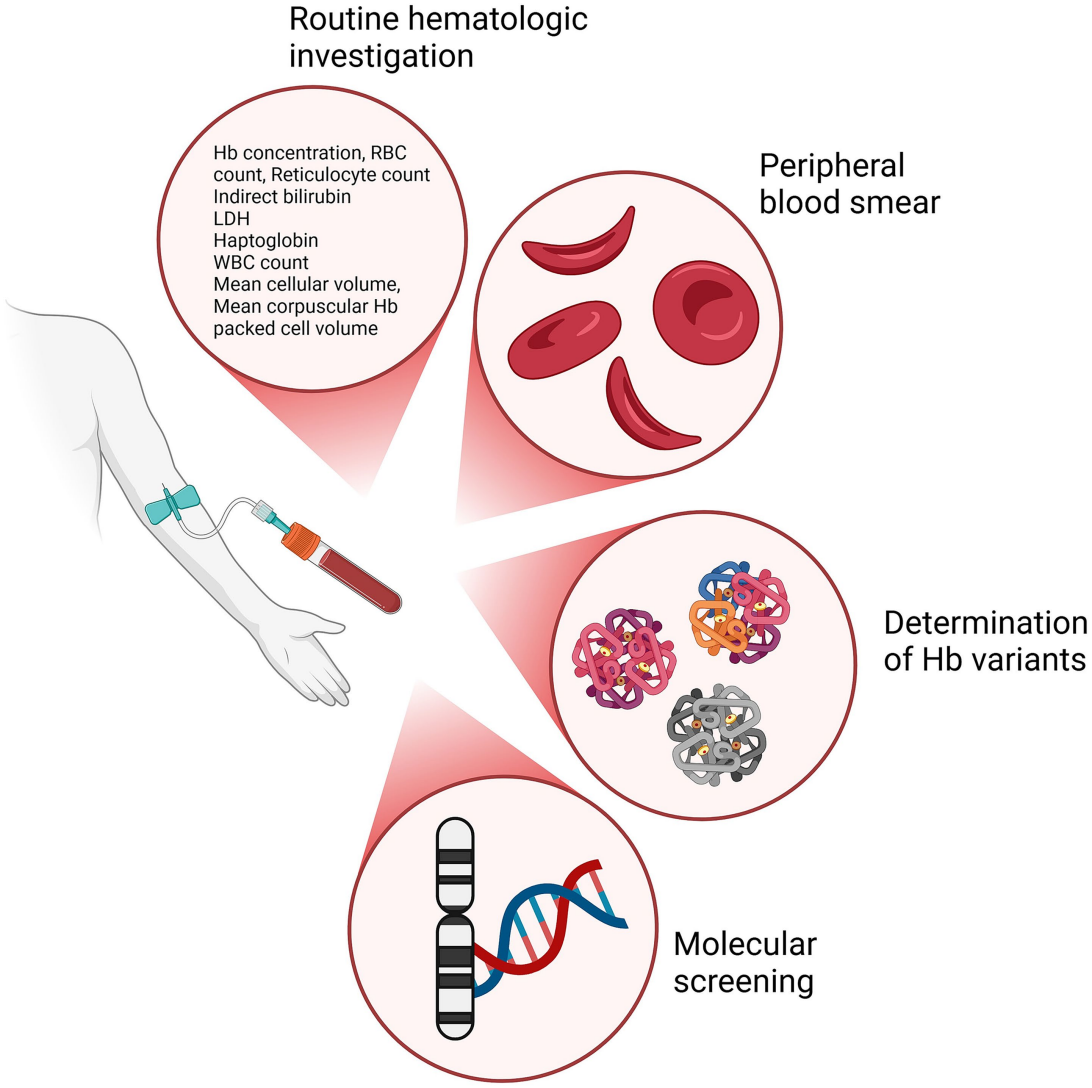
Diagnostic techniques for SCD investigation and the relative outcomes. Primary diagnostic approaches used in the evaluation of SCD: routine hematologic investigation to assess key hematological parameters, peripheral blood smear to assess the shape, size, and morphology of RBCs, determination of Hb variants identifying different Hb types, and molecular screening to identify mutations in the DNA, particularly on genes related to Hb production. Created in BioRender (2024) https://BioRender.com/c90x086.

In addition to a complete medical history and physical examination, and to adequate imaging studies to evaluate any complication, a wide range of laboratory methodologies should be applied to diagnose SCD. As mentioned above, the availability of multiple diagnostic tools for SCD and their relative appropriateness to different clinical settings may be a source of complexity not easily managed by non SCD-experts. In Table 3 and Figure 3 we summarize the main diagnostics characteristics and the analytical approach they use.

**Table 3:** Summary of diagnostic tests for hematologic evaluation, techniques, and clinical applications.

Test	Parameters measured	Technique	Timing	Ref
Routine hematologic investigation	Hb concentration, RBC count, Reticulocyte count Indirect bilirubin LDH Haptoglobin WBC count Mean cellular volume Mean corpuscular Hb packed cell volume Peripheral blood smear for RBC morphology	Automatic cell counting Staining Microscopy	In case of clinical suspect	(55–59)
Determination of Hb variants	Identification and quantification of HbA, HbS, HbF	Electrophoresis Iso-electric focusing High performance liquid chromatography Mass spectrometry	Newborn screening and in every case of clinical suspect	(26, 38, 60)
Molecular screening	Single nucleotide polymorphisms to detect specific SCD-related mutations	Multiplex PCR, ARMS, ASA, MLPA	Population-specific mutation detection Confirmation of a diagnosis (either prenatal, newborn, or adult) In case of atypical hematologic parameters In any case of clinical concern, when available data are unsure or inconsistent with clinical evidence	(31, 56, 61)

## 6 Discussion

Epidemiological data report an increased prevalence of SCD worldwide between 2000 and 2021 and a concomitant increase in mortality rate (3), underscoring the critical need for early diagnosis and management of this condition. As non-SCD-expert clinicians (especially in ERs, pediatrics, general medicine) often do not suspect SCD, it is crucial to raise awareness of key presenting features, such as anemia accompanied by pain in patients from high-risk ethnic backgrounds, and to include SCD in the differential diagnosis. Identification of SCD at an early stage and appropriate referral to a specialized center enables the proactive management of symptoms, minimizing the frequency and severity of painful crises and the onset of other complications (28, 29). Implementation of newborn screening programs as in US or UK (30, 31), currently lacking in Italy could potentially reduce the missed early diagnoses. Regular monitoring of children with SCD for early identification of complications, education of patients and their families to recognizing symptoms, understanding treatment options, and adhering to care plans can lead to a better management of the disorder (3, 4, 32).

The early identification of the people potentially at risk is challenging for clinicians who encounter SCD infrequently given to SCD’s complexity, as the clinical symptoms may overlap with other conditions (31, 33). Despite SCD’s variability, there are key clinical features that can help to raise diagnostic suspicion even among non-experts. These features include a relevant ethnicity and geographical origin (such as sub-Saharan Africa, India, the Middle East, and the Mediterranean), a family history of the disease, and hemolytic anemia, which should be not mistaken for anemia due to a chronic condition or inflammation, in a patient experiencing an acute event. Recognizing these features as part of a “core set” could prompt non-SCD specialists to consider SCD earlier in their diagnostic process. Due to the extensive migratory fluxes, SCD individuals and SCD-trait carriers are now common in European countries, including Italy (20), thus the awareness of the key clinical features associated to SCD should be shared across Italian non SCD-referral centers and especially at the territorial levels.

In addition to relevant ethnicity, family history, and hemolytic anemia, frequent or recurrent presentations of symptoms should raise high clinical suspicion to investigate SCD as a unifying diagnosis. In children, the striking example is cerebrovascular injuries that can occur upon VOCs and can lead to ischemia (21), resulting in long-term cognitive impairments (34). These symptoms may not be readily associated with SCD but should raise suspicion when occurring in at-risk populations. Transcranial Doppler screening evaluates the velocity of intracranial circulation which is a marker for risk of ischemic strokes and can be performed in specialized SCD centers, although limited access to this test is reported in Europe and Italy (21, 35).

Recurrent pain episodes of unknown origin are additional clinical features that should raise diagnostic suspicion (16). The presence of these atypical and often disparate clinical events should strengthen the suspicion of SCD and lead clinicians to consider the disorder as a part of their differential diagnoses.

Acute complications such as severe pain crises, stroke, or splenic sequestration, are clinical signs that should be considered for diagnosis in pediatric patients, while adult cases may feature more often chronic complications (21).

Education on the hallmarks of SCD and adherence to established guidelines could help to improve diagnostic accuracy and prevent recurrence of common errors in clinical practice, reducing missed or delayed diagnoses (31, 33). The lack of use of structured illness scripts to guide suspicion and diagnosis can limit the proper identification of the patient. The integration of SCD scripts “−concise summaries of core clinical features and an overview of diagnostic steps as we presented here− into clinical training, medical software, and ER triage protocols can help non-expert clinicians to recognize SCD’s warning signs and could help to standardize the initial approach to suspected SCD cases, facilitating a more structured, systematic evaluation. With such tools, non-experts could be more confident in identifying signs suggestive of SCD and could promptly refer the patient to the closest specialized center.

The establishment of clear and standardized protocols for referral based on key clinical indicators can improve the poor communication between first line health care professionals and specialized centers, to improve the access to comprehensive care thus ameliorating patient outcomes and quality of life (36).

## 7 Conclusion

Practical tips, specific training and knowledge for a rapid diagnosis of SCD are of key importance for non SCD-expert healthcare professionals. Avoidable diagnostic and referral delay continue to be burdensome consequences of this still inadequate knowledge of SCD. Practical tools, such as the proposed SCD scripts, favoring the diagnostic suspect, seem valuable and may find a place in this scenario. The diagnostic reasoning may be further strengthened by established competence about available diagnostic tools and currently available tests and their applicability in the clinical practice, to favor non-SCD expert facing with SCD diagnosis.
